# DEMENTIA FRIENDS AWARENESS BUSINESS TRAINING PROGRAM: MAKING A POSITIVE IMPACT IN SMALL ARKANSAS BUSINESSES

**DOI:** 10.1093/geroni/igad104.2649

**Published:** 2023-12-21

**Authors:** Laura Spradley, Whitney Thomasson, Robin McAtee

**Affiliations:** UAMS, Little Rock, Arkansas, United States; University of Arkansas for Medical Sciences (UAMS), Little Rock, Arkansas, United States; University of Arkansas for Medical Sciences, Little Rock, Arkansas, United States

## Abstract

The Arkansas Geriatric Education Collaborative, a HRSA-funded Geriatric Workforce Enhancement Program grant, and a Dementia Friends USA sub-licensee, provides a program to businesses called Dementia Friends at Work. This program utilizes the tenants of the “Become a Dementia Friend” program created by Dementia Friendly America©, a multi-sector movement that aims to foster “dementia friendly communities”. A one-hour training was conducted for 222 business employees, focusing on recognizing the signs of dementia, challenging the stigma about dementia, how to best communicate with individuals with dementia and their caregivers, and practical tips to make themselves and their environment more Dementia-Friendly. During the program, management and staff were asked to self-assess the physical business environment for “dementia friendliness” and review possible changes toward improvement. Pre-program and post-program evaluations were completed. The results showed that 100% of participants agreed the training program made an impact on their business or job and 75% claimed to use the knowledge gained from the program. Qualitative data gathered 90-days post training supported these results with themes emerging such as staff using new ways to navigate conversations with those expected to have dementia, being more aware of the signs of dementia, and utilization of skills learned to more effectively interact with these customers. Findings suggest that many employees were unaware of the common signs of dementia or how to recognize behaviors of those with dementia. They had also not considered learning about or using proven techniques to assist with interactions and conversations; therefore, this training was well-received and effective.

